# Blind versus open weighing from an eating disorder patient perspective

**DOI:** 10.1186/s40337-020-00316-1

**Published:** 2020-08-17

**Authors:** Franzisca V. Froreich, Sarah E. Ratcliffe, Lenny R. Vartanian

**Affiliations:** 1 School of Psychology, UNSW Sydney, Sydney, 2052 Australia; 2 School of Psychology, University of Sydney, Sydney, 2006 Australia

**Keywords:** Eating disorders, Open weighing, Blind weighing, Patient preferences, Qualitative

## Abstract

**Background:**

Weighing is a key component in the treatment of eating disorders. Most treatment protocols advocate for open weighing, however, many clinicians choose to use blind weighing, especially during the early phase of treatment. Despite considerable debate about this issue in the literature, there is no empirical evidence supporting the superiority of one weighing approach over the other. In addition, little is known about patients’ perspectives of open and blind weighing and which weighing practice they view as more acceptable and/or beneficial for their treatment.

**Methods:**

Semi-structured qualitative interviews were conducted with 41 women with a current or past diagnosis of Anorexia or Bulimia Nervosa: 26 were undergoing specialist inpatient treatment (*n* = 13 being blind weighed; *n* = 13 being open weighed) and 15 were community members who have recovered from an eating disorder. Interviews were audiotaped, transcribed verbatim and analysed thematically using framework methods. Participant demographics, clinical characteristics, weighing anxiety and weight concerns were also assessed.

**Results:**

Qualitative analyses yielded five themes: *(1) therapy engagement and progress; (2) Control and tolerance of weight uncertainty*; *(3) treatment team relationships and autonomy; (4) life outside of treatment*; and *(5) weighing practice preferences and rationale.* Participants stated that blind weighing decreased anxiety and eating disorder psychopathology (e.g., weight preoccupation) and increased treatment responsivity. For many, relinquishing control over their weight facilitated body trust and was a necessary step towards recovery. Participants found that not knowing their exact weight helped challenge their overconcern with weight. Lack of support post-discharge was identified as a major difficulty of blind weighing. Overall, the majority of participants preferred blind weighing, particularly at the early, acute stage of treatment, whereas open weighing was viewed as more suitable at later stages of recovery. Quantitative analyses found current blind-weighed patients felt significantly less anxiety around being weighed and had greater tolerance of weight uncertainty than current open-weighed patients.

**Conclusions:**

This study provided in-depth patient insights into open versus blind weighing practices. The next step for future research will be to supplement these insights with treatment outcomes gained from randomised controlled trials comparing the two weighing practices.

## Plain English summary

Weighing is a key component in the treatment of eating disorders, but the particulars of how patients should be weighed (i.e., open vs. blind weighed) is a point of debate in the field. This study explored how current and recovered eating disorder patients (*N* = 41) view and have experienced open weighing and blind weighing in treatment. Analysis of interview data found that the majority of participants preferred blind weighing, particularly at the acute stage of treatment, whereas open weighing was viewed to be most suitable at lager stages of recovery. Participants thought that blind weighing decreased anxiety and eating disorder symptoms and as a result, increased their ability to effectively engage in their treatment. Survey data confirmed that current blind-weighed patients felt significantly less anxiety around being weighed and were more comfortable not knowing their weight than current open-weighed patients. The results of this study provide important insights into eating disorder patients’ experiences with open and blind weighing practices and offer options for future research.

## Background

In-session weighing is a prominent feature of eating disorder treatments, partly because of the need to monitor the physical safety of some patients [1]. There are two main approaches to weighing, “open weighing” and “blind weighing”. Open weighing involves in-session weighing whereby the therapist and the patient check the patient’s weight together. The number on the scale is read out loud to prevent avoidance of knowledge of weight. The weight is then plotted and interpreted on an individualised weight graph [2, 3]. Open weighing is grounded in the belief that repeated exposure to one’s weight reduces patients’ fear of being weighed and/or knowing their weight, and that open weighing provides an opportunity to modify the “broken cognition” many eating disorder patients have that consuming calorie-dense foods leads to exponential and uncontrollable weight gain [1, 4]. Conversely, blind weighing involves the therapist not sharing the patients’ weight with her or him. When patients are blind weighed, they are usually asked to step on the scale backwards and the weight is not explicitly discussed [3]. The rationale for blind weighing includes the desire to minimise anxiety and distress that may result from patients seeing their weight (especially when it increases), reduce the patients’ focus on the specific number on the scale, and expose patients to weight uncertainty [3, 5].

Two of the most widely established therapies for eating disorders, cognitive-behaviour therapy (CBT) and family-based treatment (FBT), both advocate for open weighing [1], yet specific and compelling evidence demonstrating that open weighing is preferable for patient outcomes is lacking. Moreover, weighing practices by eating disorder professionals, even those who endorse a CBT or FBT orientation, is far from unanimous: only about half of eating disorder clinicians subscribe to open weighing whereas the other half report using blind weighing procedures [3]. Forbush and colleagues [3] failed to find a significant association between endorsement of CBT or FBT and use of open weighing. Instead, their findings suggested that clinicians’ decision to practice open or blind weighing depended on patient diagnosis and stage of treatment. Specifically, clinicians were more likely to practice blind weighing if patients were in the acute stages of treatment (and thus perhaps less stable or less committed to treatment) and/or if patients’ cognitive or emotional functioning appeared to be impaired from malnourishment [3].

In addition to a lack of empirical evidence supporting the superiority of one weighing approach over the other, little is known about patients’ perspectives of open and blind weighing and which weighing practices they view as more acceptable and/or beneficial for their treatment. Poor treatment engagement and premature termination of treatment are particularly common problems for eating disorder patients, with dropout rates ranging between 20 and 51% and 29–73% in inpatient and outpatient settings, respectively [6]. Given that weighing can be experienced as an anxiety-provoking event, it could conceivably contribute to difficulty engaging with the treatment. Thus, understanding patients’ perspectives about weighing practices, and integrating their views into current treatments, could help improve client engagement and retention and, as a consequence, treatment outcomes in this population.

The present study used qualitative methodology to explore how current and recovered eating disorder patients view and have experienced open weighing and blind weighing in treatment. Eating disorder inpatients were chosen because they are weighed at a greater frequency than are outpatients (daily to twice weekly, compared to weekly, respectively) [7] and thus are most affected by weighing practices. To complement inpatient views, recovered community members were included to provide post-treatment perspectives that are less affected by the ambivalence which characterises eating disorders as well as to improve understanding of the long-term effects of open and blind weighing. The overall aims of this study were to elucidate: (1) patients’ acceptance of open weighing and blind weighing; (2) the factors that shape patients’ preferences for a particular approach to weighing; and (3) the impact that open weighing and blind weighing may have on therapy engagement and treatment outcomes including weighing anxiety, dysfunctional beliefs about weight, and intolerance of weight uncertainty.

## Method

### Participants

#### Current patients

Current female patients were recruited from two specialised eating disorder inpatient units: one which routinely blind weighs patients (blind weighing group; *n* = 14), and one which routinely open weighs patients (open weighing group; *n* = 13). To be eligible for this study, participants had to be at least 18 years old, diagnosed by the treating psychiatrist with anorexia nervosa (AN), bulimia nervosa (BN), or other specified feeding or eating disorders (OSFED), and have completed two or more weeks of inpatient treatment and hence be familiar with the weighing protocol. All inpatients (100%) approached by the study coordinator (FF) agreed to participate. One blind weighed patient was excluded from analysis because she felt unwell and terminated the interview early.

#### Recovered participants

Recovered female community members (*n* = 22) were recruited via expression of interest flyers posted on the social media platform of national eating disorders support organisations, the university’s social media pages, the National Eating Disorder Collaboration’s website, and dissemination via clinician emailing-lists. The study’s advertisement indicated that the researchers were looking for women previously diagnosed with an eating disorder who were currently recovered. In this study, “recovered” was defined as no longer meeting the Diagnostic and Statistical Manual of Mental Disorder, Fifth Edition (DSM-5) [8] diagnostic criteria for an eating disorder. To screen for potential threshold or subthreshold eating disorder diagnoses, all recovered participants who expressed interest in the study were assessed using the Eating Disorder Diagnostic Scale – DSM-5 version (EDDS) [9]. Some respondents indicated not knowing their weight (EDDS item 19). For these cases a conservative approach was taken by treating them as if they met the weight criterion for diagnosis of AN; that is, it was assumed that they had a BMI ≤ 18.5. Six recovered participants were excluded because they met EDDS criteria for atypical AN (*n* = 1) and BN (*n* = 1) or did not respond to subsequent contact from FF (*n* = 4), resulting in 16 (72.7%) interviews. In addition, one participant was excluded after completing the interview because of incoherent and incongruent responses.

Recruitment continued until data saturation (i.e., no new information after three consecutive interviews) [10]. Ethical approval for all aspects of the study was obtained by the university’s and the two hospitals’ ethics committees.

### Procedure

At the initial screening stage, FF who had no pre-existing relationship with any of the participants, introduced the nature and purpose of the study to interested participants. After obtaining written informed consent, a one-off face-to-face interview was conducted with participants in the inpatient units. For recovered participants, the interview was conducted over the telephone. The mean duration of current patients’ and recovered participants’ interviews were 10.69 min (*SD* = 2.45) and 26.86 min (*SD* = 8.93), respectively. Following the interview, participants were asked to complete a short survey online. Participants were debriefed by FF and reimbursed $10 for their participation.

### Qualitative data collection

A semi-structured interview protocol (see Additional files 1 and 2) was purposely designed for this study in consultation with an experienced qualitative psychology researcher. The questions were guided by the study’s research aims and existing hypotheses from the literature about the benefits and drawbacks of open and blind weighing. Parallel interview protocols were developed for current patients (open weighing and blind weighing) and recovered participants. During the interview, participants were asked about their experience with weighing in the context of the treatment for their eating disorder and any particular benefits and drawbacks they identified. Specifically, questions focused on if and how the weighing practice that they had experienced had affected their progress in treatment, motivation with treatment, ability to comply with meal plan, preoccupation with weight, anxiety around being weighed, and eating disorder symptomatology.

### Quantitative measures

Eating disorder pathology in recovered participants was assessed using the 23-item EDDS which captures general eating disorder symptoms, as described in the DSM-5 [8]. Adequate criterion, predictive, and convergent validity, internal consistency, sensitivity, and test-retest reliability were documented for the version of the EDDS developed for DSM-IV [11, 12]. Although validation of the EDDS DSM-5 version is still underway, preliminary data regarding suggest high agreement of EDDS diagnoses with interview measures [13].

Participants’ current weight concerns were assessed with five items developed for this study: Intolerance of weight uncertainty (1 item; “I need to know how much I weigh”), weight preoccupation (1 item; “I am preoccupied with my weight”), and weight importance (3 items; e.g., “My weight is very important to me”). Items were rated on a six-point scale from 1 (*Never*) to 6 (*Always*). Weight importance items were averaged, with higher scores indicating greater perceived importance of weight. Cronbach’s alpha for the weight importance subscale was .94. In addition, a global weight concern score was calculated by averaging all five items. Cronbach’s alpha for the global score was .96.

Weighing anxiety was measured using a visual analogue scale from 0 (*Not at all anxious*) to 100 (*Extremely anxious*). Current patients were asked to rate how anxious they felt being weighed in the context of treatment. Recovered participants were asked to rate how anxious they would feel if they were weighed right now.

All participants reported their age and ethnicity. Recovered participants also reported their height and weight (if known), which were used to calculate their body mass index (BMI [kg/m^2^]). For current patients, height and weight was retrieved from their medical records.

### Data analyses

Questionnaire data were analysed with SPSS version 24.0. Audio-recorded interviews were transcribed verbatim and NVivo 11 was used for thematic analysis [14]. Thematic analysis followed the five main steps of Spencer and Ritchie’s [15] framework method: (1) *Familiarisation with the data:* FF conducted all interviews, cross checked each transcript with the audio-recording and read each transcript several times; (2) *Creating a thematic framework:* a preliminary framework was based on independent analyses of 20% of transcripts by FF and a co-author with qualitative expertise (SR). Data were independently organised using themes and subthemes. Differences in interpretations of data were discussed until consensus was reached. (3) *Indexing:* adhering to the thematic framework, FF and SR independently coded all transcripts using NVivo11. Any new themes or questions that emerged during this process were collaboratively discussed and revisions made correspondingly. To ensure methodological rigour, all transcripts were then independently cross-coded by FF and SR and discrepancies discussed and resolved with input from LV. (4) *Charting:* themes and supporting quotes from each transcript were entered into a framework matrix. The matrix was composed using Microsoft Excel as a computerised qualitative data analysis tool [16], with participants as rows and themes as column. (5) *Mapping and Interpretation*: the framework matrix was analysed within and across themes and participants to identify patterns and relationships.

## Results

### Descriptive statistics

Table 1 presents the demographic and clinical characteristics for all three samples. Blind weighed patients and recovered participants did not differ in their mean age or BMI, but open weighed patients were significantly younger and had lower BMIs than did both blind weighed patients and recovered participants.

**Table 1:** Participant Demographic Characteristics

	BW *M* (*SD*)	OW *M* (*SD*)	Recovered *M* (*SD*)	Total *M*
*N*	13	13	15	41
Age	30.08 (10.60)	20.62 (2.59)	29.27 (10.21)	26.65
BMI	19.93 (3.56)	15.57 (3.30)	19.8 (3.54)	18.43
	n (%)	n (%)	n (%)	n (%)
Diagnosis				
AN	12 (92.3%)	12 (92.3%)	11 (73.3%)	35 (85.4%)
BN	1 (7.7%)	–	2 (13.3%)	3 (7.3%)
OSFED	–	1 (7.7%)	2 (13.3%)	3 (7.3%)
Treatment Exposure				
Open Weighing	–	5 (38.5%)	3 (20.0%)	8 (19.5%)
Blind Weighing	4 (30.8%)	–	2 (13.3%)	6 (14.6%)
Both	9 (69.2%)	8 (61.5%)	8 (53.3%)	25 (61.0%)
Other	–	–	2 (13.3%)	2 (4.9%)
Preference				
Open Weighing	1 (7.7%)	11 (84.6%)	3 (20.0%)	15 (36.6%)
Blind Weighing	12 (92.3%)	1 (7.7%)	11 (73.3%)	24 (58.5%)
Undetermined	0 (0%)	1 (7.7%)	1 (6.6%)	2 (4.9%)

*Note. BW* Blind weighing, *OW* Open weighing, *BMI* Body mass index, *AN* Anorexia nervosa, *BN* Bulimia nervosa, *OSFED* Other specified feeding or eating disorder

### Qualitative findings

Qualitative analyses yielded five inter-related themes: (1) therapy engagement and progress; (2) control and tolerance of weight uncertainty; (3) treatment team relationships and autonomy; (4) life outside of treatment; and (5) weighing practice preferences and rationale. Themes found were represented among all three groups, with blind weighed patients and recovered participants expressing concordant views toward weighing practices (i.e., a preference for blind weighing), whereas open weighed patients expressed stronger preference for open weighing than blind weighing. Illustrative quotes are presented in Tables 2, 3, 4, 5 and 6. Because the majority of participants (61.0%; *n* = 25) had been exposed to both open and blind weighing across their treatment history, their responses were grouped together and are described with respect to the particular weighing practice that was being discussed rather than presenting the results separately by participant groups.

**Table 2:** Illustrative Participant Quotations for Theme 1: Therapy Engagement and Progress

Subtheme		Illustrative participant quotations
1.1 Weighing experience	BW	*“There is a lot less anxiety around [being weighed] ... I just kind of get it over and done with. Because I’m not, dreading it really, since I’m not seeing it. I don’t love it, but I’m not dreading it the same way I was.”* (Reduced anxiety, BW109) *“I feel a bit anxious for a bit, but then I’m like, ‘whatever’, and just go do something.”* (Reduced anxiety, OW112) *“[BW] was quick and painless, you just kind of get up, get weighed go back to bed”* (Reduced anxiety, R315)
	OW	*“I’m more stressed before weighing days, and then the day I’m being weighed, thinking about it more probably … I get anxious, tossing and turning, I think about it, I catastrophise like ‘oh my god it’s gone up 10 kg it’s probably gone up this much, it’s gonna [*sic*] be this weight. If it’s up this high I’ll do such and such, if it’s below this I’ll do such and such.”* (Increased anxiety, OW211) *“Yeah I think that weighing days were definitely the worst ones … you’d wake up and you’d know that you have to be weighed and it was, you know when you get that pit in your stomach where you kind of want to throw up, it was like that.”* (Increased anxiety, R315)
1.2 Therapy engagement	BW	*“I think sometimes [BW] can exaggerate or catastrophise what your thoughts are because you don’t have any evidence, like you can eat something you are scared of and think ‘Oh my god it’s going to make me gain 5 kg’ and then you don’t really have any way of proving it wrong? Which I think can be hard. But obviously, I know like if I said that, my treatment team would happily provide me evidence that it didn’t make me gain a ridiculous amount of weight. But I think that can be hard.”* (Distorted cognitions about weight gain, BW113) *“Not knowing [my weight helps me comply with my meal plan], because then I’m not kind of obsessed about a number all the time and I’m just doing what I have to do really.”* (Increased meal plan compliance, BW102) *“I was like well I don’t know my weight. Yes it’s terrifying but at the same time it meant that I focused on getting through my meal and focused on my psychology sessions and the group therapy that we did instead of ‘oh my weight was this today’ … I guess it was just one less thing that I had to deal with.”* (Increased therapy engagement, R315) *“The more you check something the more it might be on your mind and by not checking my weight it was something that became less and less pronounced in my day to day [routine].”* (Reduced weight preoccupation, R310)
	OW	*“The longer I get into the admission and the more weight I gain, I get more preoccupied with it [my weight] and I get more anxious.* (Increased weight preoccupation, OW212) *“Obviously if it’s gone up more than I’m comfortable with I’m less likely to comply [with my meal plan]. The other week I only drank supplements for the whole day, and refused to get out of the bed other than for meals because of the number jumping up”* (Increased resistance to meal plan, OW211) *“Being open weighed definitely impacted on my motivation and ability to stick to meal plan. It made me work “against” therapy and made it harder to comply.”* (Increased resistance to meal plan and reduced motivation, R311)
1.3. Progress	BW	*“Initially, it was worse...not knowing, predicting the worst, I guess. But then after a couple of weeks, you kind of got used to it and now it’s a bit more peaceful.”* (Early adjustment difficulties, OW213) *“[BW] keeps your mind on track of the end result which is getting better. It doesn’t leave you with all these preoccupations and distractions.”* (Reduced preoccupation, OW110) *“[BW] was in many ways the only way that I had to learn to not put all the emphasis into my own weight. That, by not knowing my weight, I had to learn to be comfortable not knowing it, and therefore not emphasizing it.”* (Reduced weight importance, R306)
	OW	*“If it [my weight] goes up I tend to get very anxious and tend not to want to do the right things in my treatment, and I want to go home.”* (Treatment resistance, OW212) *“I think like weighing for me was the thing that probably kept me back the most when I decided to recover.”* (Barrier to recovery, R315) *“I think it’s important for us to see the weight go up because it’s what’s needed, and it will help rationalise those thoughts that if you eat something, you’re not going to magically gain 10 kg, like most of us think.”* (Challenging of distorted weight beliefs, OW209)

*Note*. BW: Quotes relating to blind weighing; OW: Quotes relating to open weighing; BW# blind weighed inpatient; OW#, open weighed inpatient; R#, recovered participant

**Table 3:** Illustrative Participant Quotations for Theme 2: Control and Tolerance of Weight Uncertainty

Subtheme		Illustrative participant quotations
2.1 Need of control	BW	*“Especially because once you’re in here [treatment], you have no control … and sometimes you just want to hold onto one thing. And not just another thing that you lose.”* (Need for control, BW111)
	OW	*“I would never be blind weighed. … what I care about is numbers. If I don’t know the number, then I don’t feel in control … It’s like the one thing you can control … you don’t have control over how much petrol is going to be, if there’s going to be red lights or green lights, you don’t have control if there’s going to be crashes. So, the one constant thing I can control is what I put in my body and what my weight will be.”* (Need for control, OW211) *“If you knew the number, there was a kind of disorder mindset where it had to keep going down and if it wasn’t, then that wasn’t okay.”* (Need for control over weight, R310) *“[If I had been OW], I don’t think I could’ve been able to keep going once I saw that I had hit a certain number.”* (Acceptable number, R316)
2.2 Tolerance of Weight Uncertainty	BW	*“[BW] is definitely good that you don’t have that preoccupation around the number, but still there’s a worry that obviously you constantly have about how much you are weighing and how much weight you are gaining doing this program.”* (Intolerance of weight uncertainty, BW110) *“I’ve been in other clinics where I have had to see my weight every week and it’s been traumatising, and it sucks. And like, I find ignorance is bliss.”* (Reduced weight-related anxiety, BW113) *“I think it’s just one less thing to worry about... I think that it’s very easy to fixate on individual numbers and figures whereas what I think is most important in here is the overall trend... and I just find it less stressful not having another number to deal with in my head, as long as I know that my weight is going up, that’s all I really need to know.”* (Reduced weight-related anxiety, BW107) *“For me personally, there is a bit less fear around weight change at the moment because I’m not going to see the weight change. Like, I just kind of have to accept whatever’s happening and just roll with it, since I can’t see it.”* (Reduced weight-related anxiety, BW109) *“I don’t think normal people would like, would weigh themselves like every day. It’s not something they think about, they might just do it.... I would, probably like to just never have to weigh myself again, and not care about it. And just be healthy.”* (Normalising lack of control over weight, BW106) *“Having to relinquish that control [over my weight] was terrifying but was also a huge relief and it really helped me to learn to be at peace with my body and to trust [my body].”* (Increased body trust, R310) *“When I think about what I want my life to be in recovery, I don’t want to know, and I want to be okay not knowing, because to me, that sounds like a more free life to me, than one where I have to know.”* (Tolerance of weight uncertainty, R316) *“I think it [BW] kind of sets the platform for you to then be able to then take the focus off the weight when you go back into the real world and to be able to go ‘actually it [not knowing my weight] hasn’t killed me, it’s not the worst thing in the world, and it’s okay.”* (Tolerance of weight uncertainty, R315) *“I don’t weigh myself now. I am happy knowing that I look and feel healthy.* “(Tolerance of weight uncertainty, R304)
	OW	*“I couldn’t cope [not seeing the number].”* (Inability to cope with weight uncertainty, OW211)
2.3 ED-Self vs. Healthy-Self	BW	*It annoys me [not seeing my weight], but at the same time, that’s my eating disorder that it annoys, but like, for my healthy self, it’s good.”* (Need for control is ED-driven, BW101) *“[BW] takes control away from the eating disorder rather than the person.”* (Need for control is ED-driven, BW109) *“I feel like my eating disorder was very much like control, and about controlling... just my life. And it was, as soon as I let go of the control of the scales … it was easier to just let that part of me go. So that control... that inner voice that had control over me just went away.”* (Need for control is ED-driven, R302) *“I do know of other people … that did see their number … they would feel really shit about it but still maintain ‘oh no it’s important that I see it because I know where my weight is’ and I was like ‘is it important for you or is it important for your eating disorder?’”* (Need for control is ED-driven, R310) *“As soon as I stopped looking at the scales, I had to stop thinking about the number, and it stopped controlling me … then I learnt how to eat better...and treat my body well, and it wasn’t about losing weight or gaining weight because I just let it go.”* (Increased body trust, R311)
2.4 Food-Weight Associations	BW	*“I guess because it’s over the week, and we have three weigh-ins. I don’t know when I gained the most weight or anything. I just know the overall trend so that means I can’t pinpoint to ‘oh my god that was that meal!’ BW helps not associating certain weight gains with certain meals.”* (Challenged food-weight associations, BW105)
	OW	*“I just think it [OW] would be so much harder to like complete meals or like keep going forward because the thoughts in your head, it’s saying like ‘Oh, if I eat this I’m going to gain weight” and then you’re actually seeing it happen, it’s like reinforcing it all.’* (Maintenance of food-weight associations, BW113) *One part of me wants to know [my weight] I guess so I can micromanage things, and be like, ‘Okay, what do you think would have tipped it over?’”* (Maintenance of food-weight associations, OW213) *“If numbers had been there and it had been tracked closely I think for me personally it would have been really hard to separate those really intertwined concepts of food and weight.”* (Maintenance of food-weight associations, R310)

*Note*. BW: Quotes relating to blind weighing; OW: Quotes relating to open weighing; BW# blind weighed inpatient; OW#, open weighed inpatient; R#, recovered participant

**Table 4:** Illustrative Participant Quotations for Theme 3: Treatment Team Relationships and Autonomy

Subtheme		Illustrative participant quotations
3.1 Treatment autonomy	BW	*“I found it [BW] very like punitive in some ways. Like I don’t know I’m an adult and I just felt like I was a child being like weighed backwards and then kind of like in trouble if it wasn’t the outcome that was hoped for or something like that.”* (Lack of treatment autonomy, R316)
	OW	*“You can see how your weight is going up steadily, and your meal plan’s working. Whereas if they were to just increase your meal plan and you’re like, ‘why?’, it kind of answers some of those questions.”* (Treatment progress feedback, NW213) *“I feel like knowing the number includes you in the conversation with the treatment team.”* (Inclusion in treatment conversations, NW207) *“I can’t see any benefits [to OW] other than feeling a little more autonomous and “normal”.”* (Increased treatment autonomy, R304) *“It was good to feel like I had some autonomy and was allowed to see it although I guess I could equally be autonomous choosing not to see the weight right?”* (Role of autonomy in treatment, R304)
3.2 Trust in treatment team	BW	*“I like that they are in control of my weight and I can’t know it. They just showed you what your [weight trend] was doing and I liked that, if you did it with someone you trusted.”* (Importance of trust in treatment, N101) *“In the beginning I hated it … I always used to think like, ‘oh no, they’re lying, they’re lying, they’re lying, they’re lying’ … but I think after coming to [BW facility] now for a year, and I know now that my relationship with [psychiatrist and psychologist], I know that I can trust them, like a 100%. Now it’s kind of nice as well to give the control over to someone else.”* (Importance of trust in treatment, N112) *“I think those are the two things you really lose sight off when you’re unwell you, there’s so much control and so little trust and [BW] kind of just turns it on its head.”* (Importance of trust, R310) *“I previously had a psychologist that completely lied to me about my weight so it got -- like, a really long time and just, it’s made me petrified. She used to say, ‘No I won’t let you look.’ And I would say, ‘Well, is it below this number?’ And she would say, ‘Yes.’ And then one day I found out that she had been lying all that time.”* (Need for honesty, NW209)
3.3 Strategies for Enhancing Autonomy and Trust	u/s	*“I wish we had the ability to kind of... Talk to somebody about it [best weighing practice], without being shut down.”* (Involvement in treatment decision-making, N108) *“I feel that the way they do it here is quite good with giving you a choice...challenging ‘Hey do you want to try and see your number and have therapy around that while you’re here?”* (Choice, NW213) *“During therapy, I knew my weight at some stages and then I didn’t know it at others. It depended in what the therapist thought was best. I sometimes felt like I wasn’t being asked, I wasn’t being treated like a “normal person” who could be trusted and could take responsibility of it. Perhaps it would have been good to have that discussion with me and see if it was a “recovery day” where I felt capable of seeing it or not.”* (Involvement in treatment decision-making, R304)

*Note*. BW: Quotes relating to blind weighing; OW: Quotes relating to open weighing; BW# blind weighed inpatient; OW#, open weighed inpatient; R#, recovered participant

**Table 5:** Illustrative Participant Quotations for Theme 4: Life Outside of Treatment

Subtheme		Illustrative participant quotations
4.1 Weighing, weight exposure, and discharge support	BW	*“While being here it’s better not to know but then I’m going to go home and I’m going to find out the number and then, like, what are we going to do?”* (Lack of support post-discharge, BW103) *“[BW] didn’t help you deal... with weight... They’d just let you out and you’d go home and you’d be a higher weight and you’d have to deal with it.”* (Lack of support post-discharge, OW205) *“I feel like sometimes you’re faced with it … unexpectedly like at a doctor’s appointment … Then it’s like such a big difference from what you last saw. And then it’s just like... It ruins everything.”* (Weighing is inevitable, BW108)
	OW	*“I prefer to know, so that I can get help dealing with it. Because when I’m at home, if I get weighed, I have to so often get weighed, I need to know how to deal with it.”* (Support dealing with weight exposure, OW205)
4.2 Weight change awareness	BW	*“Not knowing my weight doesn’t mean that like, I’m not, like conscious, that I’m not aware that it’s changing.* (Weight change awareness without numbers, BW101) *“Even if you can’t see [your weight] like you know it’s still happening, and you can be told that you weight is trending upwards or it’s going in the right direction or whatever but, it doesn’t reinforce [the food-weigh associations] as much in your head.”* (Weight change awareness without numbers, BW113) *“I’m aware I’m gaining weight without seeing the number …*. *I guess when you leave, if you did weigh yourself, it could be a bit of a shock. But to be honest, they give us enough feedback I kinda [*sic*] know where I would sit.”* (Weight change awareness without numbers, BW105)

*Note*. BW: Quotes relating to blind weighing; OW: Quotes relating to open weighing; BW# blind weighed inpatient; OW#, open weighed inpatient; R#, recovered participant

**Table 6:** Illustrative Participant Quotations for Theme 5: Weighing Practice Preferences

Subtheme		Illustrative participant quotations
5.1 Open Weighing	OW	*“I like to see the number … Like I can’t sit comfortably if I don’t know.”* (Need for Control, OW201) *“I think it’s a way I can see progress, and it gives a sense of comfort I guess, in terms of just knowing what your body is doing.”* (Progress feedback, OW207) *“I’m finding it [OW] more useful because then I can kind of rationalise that you know that you know I gained like 2 or 3 k in a week when that’s not the truth. So, it kind of helps me challenge the eating disorder a bit by knowing that you’re wrong, because I only gained this much. But I always prefer to know.”* (Cognitive challenging, OW210) *“Blind weighing was the worst because it gave me that panicky feeling of not knowing.”* (Intolerance of weight uncertainty, R309)
5.2 Blind Weighing	BW	*“I think not knowing makes treatment, I think it, sort of takes it away from the weight, and it’s just what your body is supposed to be.”* (Reduced weight importance, BW106) *“I think it’s just one less thing to worry about... I think that it’s very easy to fixate on individual numbers and figures whereas what I think is most important in here is the overall trend.”* (Reduced anxiety and preoccupation with weight, BW107) *“I think it [BW] kind of sets the platform for you to then be able to then take the focus off the weight when you go back into the real world and to be able to go “actually it hasn’t killed me it’s not the worst thing in the world and it’s okay.”* (Exposure to weight uncertainty, R315) *“[not seeing my weight] certainly helps me to make progress and be much healthier on a day to day basis”* (Progress towards recovery, R311)
5.3 Blind weighing adaptations	BW	*“At [BW treatment facility] they had a weight graph … it didn’t have numbers, it just showed you what your weight was doing...and I like that … it’s … a little bit of exposure.”* (Weight Graph, BW101) *“BMI banding gave me some safeguards … I would know where I might be sitting … I think it’s kind of exposure [to weight gain] but without exposure to the actual kilo amount. Banding for me gave me leeway of the flexibility of being able to move up and down, so 100 g wouldn’t send me into panic, you know, if I moved from, I don’t know, 40 kg to 40.1 kg then knowing that would send me spiralling into anxiety … but not seeing those really small incremental changes, I think reduced … the emphasis that I had on my weight, but the bands still meant that I didn’t feel like I was out of control.”* (BMI Banding, R306)
5.4 Stage of Treatment	BW	*“I think during the weight gain process, [blind weighing] definitely makes things a lot easier. Like obviously it’s not completely, stress free... But … if I were continuing to see my weight go up I’d be a lot more stressed out. So... I think it helps a little bit with the motivation to keep following my meal plan, and stuff like that, and comply.”* (Acute stage, BW108) *“I would say for girls who have first come in that, perhaps blind weighing is more beneficial … Because the eating disorder voice is so strong, and any upwards movement it doesn’t matter if it’s 200 g or 2 kg, I think that that would completely freak you out and you’d be a mess for that whole day I’d imagine after weighing. And your head would be screaming at you to not follow the program.”* (Acute stage, OW207)
	OW	*“[OW] definitely not early on. Perhaps when you start being on the recovery road you may discuss it with the clinician and together decide what would be best. But even when I thought I was ready later it turned out that I wasn’t so maybe patients wouldn’t have that insight …*? *Perhaps when fear of weight change is gone you can start seeing the weight …*” (Best timing for OW, R304)

*Note*. BW: Quotes relating to blind weighing; OW: Quotes relating to open weighing; BW# blind weighed inpatient; OW#, open weighed inpatient; R#, recovered participant

#### Theme 1: therapy engagement and progress

Within this theme, three subthemes were identified.

##### Weighing experience

Participants’ experience of being weighed differed depending on whether they saw or did not see their weight when stepping on the scale. When open weighed, participants described weighing days as anxiety-, fear-, and stress-ridden. Negative emotions and thoughts about being weighed often started the day prior to being weighed, impacting participants’ sleep and triggering urges to engage in compensatory and self-harm behaviours. Similarly, following open weighing, participants reported experiencing distress, low mood, rumination, and worry which did not improve until the end of the day. In contrast, blind weighing was described as quick and routine-like. Leading up to weighing, participants expressed little to no negative thoughts about their weight. Although anxiety was experienced by many immediately before stepping on the scale, participants appeared to be able to recover quickly and refocus on their treatment.

##### Therapy engagement

Participants noted that weighing practices influenced their motivation, meal plan compliance, compensatory behaviour engagement, rumination, and therapy engagement, especially on weighing days. Open weighing was said to decrease motivation (*n* = 14), increase resistance to meal plan compliance (*n* = 23), and increase engagement in compensatory behaviours (*n* = 22). Open weighing was further thought to trigger increased rumination and preoccupation with weight (*n* = 22) which was reported to negatively impact on therapy engagement immediately following weighing. In contrast, blind weighing was reported to maintain motivation towards recovery and treatment (*n* = 5), improve meal plan compliance (*n* = 15), and reduce urges to engage in compensatory behaviours (*n* = 10). However, not knowing one’s exact weight in blind weighing was noted to lead to catastrophic cognitions about possible weight gain because of the lack of evidence to disconfirm one’s distorted beliefs (*n* = 11).

##### Treatment progress

The majority of participants identified open weighing as a hindrance to recovery. Being exposed to one’s weight was said to increase anxiety (*n* = 16), trigger eating disorder behaviours in an attempt to control one’s weight (*n* = 17), and maintain both the patient’s and treatment team’s focus on weight (*n* = 7). Further, participants found that exposure to their weight fuelled their ambivalence towards recovery and increased resistance towards treatment (*n* = 11). Although participants noted these adverse effects of open weighing, many valued being exposed to their weight while in treatment where they had access to support from their treatment team (as opposed to at home post-discharge where they had less support; *n* = 6). Participants also acknowledged that exposure to the number could help disconfirm irrational beliefs about the relationship between food intake and weight gain (*n* = 10), challenge disturbances in self-perceived weight gain (e.g., feeling that one is gaining weight when body weight is stable), and help with weight acceptance (*n* = 13).

Blind weighing was largely seen has having a positive impact on participant’s progress towards recovery (*n* = 32). Initially, participants struggled to adjust to the blind weighing protocol, identifying feelings of lack of control, uncertainty over their weight and catastrophic cognitions about possible weight gain to be most challenging (*n* = 11). However, participants also reported that, over time, they experienced reduced preoccupation with weight (*n* = 22). They described being able to “let go” of thoughts about weight more easily because they did not have any evidence to reinforce their fears. Participants also found blind weighing to help challenge the importance placed on weight and the need to control their weight (*n* = 22). Participants explained that blind weighing took the focus away from weight and helped them “direct their attention elsewhere” (BW107),^1^ especially towards other aspects of recovery. Participants also commented that not knowing their weight forced them to stop determining their self-worth based on weight. Additionally, blind weighing was seen to promote a recovery mindset supporting movement towards recovery goals instead of eating disorder goals (*n* = 10).

#### Theme 2: control and tolerance of weight uncertainty

Within this theme, four subthemes were identified.

##### Need for control

The need for control was a phenomenon noted in all three samples and raised at various points throughout the interviews. Specifically, not seeing one’s weight was linked to not being in control. Participants who endorsed open weighing reasoned that they “don’t feel in control” (OW211) unless they are privy to their exact weight information (*n* = 8). Treatment was perceived to threaten participants’ sense of control because they were not able to engage in disordered eating behaviours such as dietary restriction, self-induced vomiting, or excessive exercise. In this context, not knowing one’s weight was seen as yet another form of losing control. Further, participants felt the need to monitor their weight to ensure that it was lower than or did not exceed what they perceived to be an “acceptable weight” each time they were weighed (*n* = 8).

##### Tolerance of weight uncertainty

When participants were blind weighed, they commonly struggled with weight uncertainty and feelings of lack of control (*n* = 16). For some participants, being kept in the dark regarding their weight while their treating team knew their weight was the most challenging aspect of treatment (*n* = 4). In spite of these challenges, participants saw the benefits of blind weighing in that it relieved some of the anxiety associated with weight change, reduced their preoccupation with weight and, as a result, allowed them to focus on recovery instead of the number on the scale (*n* = 16). Furthermore, tolerance of weight uncertainty was viewed as something worth striving for because it was regarded as a marker of recovery (*n* = 10). In fact, many recovered participants preferred not to weigh themselves post-treatment, commenting that the need to control their weight was associated with their eating disorder (*n* = 12). Instead, recovered participants preferred to rely on non-weight related markers to determine their level of “health” or “well-being,” including the fit of their clothing, reflection in the mirror, or food consumed (*n* = 8).

##### ED-self vs. healthy-self

Participants referred to the “Eating Disorder-Self” and “Healthy-Self” as two distinct mindsets which influenced their need for control and knowing their weight (*n* = 21). The Eating Disorder-Self (ED-Self) was described as a separate, disordered self that kept participants trapped in the obsession of controlling their weight. The need to know one’s weight (and, therefore, feeling in control of one’s weight) was viewed as intrinsically connected to the eating disorder and hence pathological. Thus, when participants identified their ED-Self to be more dominant than their Healthy-Self, there was a preference for open weighing. The Healthy-Self, on the other hand, was described as the side of the self which desires recovery and to “do the right thing,” including giving up control over weight. Blind weighing was perceived as an opportunity to practice tolerating weight ambiguity and normalise the lack of control over weight. Amongst recovered participants, there was also specific reflection on how blind weighing helped rebuild body trust. Rather than eating (or not eating) in response to the number on the scale, participants reported increased trust and reliance on their body’s natural hunger and fullness signals. Furthermore, participants explained that blind weighing helped change the rationale for food and eating from sources for controlling one’s weight and shape to sources for looking after one’s body.

##### Food-weight associations

A subset of participants reflected on how different weighing practices affected their beliefs about the relationship between ingestion of specific foods and their weight. When participants saw their weight, they attributed weight changes to specific foods previously consumed (*n* = 6). If, for example, participants had eaten a “feared food” and experienced increased weight, the weight increase would be attributed to the feared food, reinforcing dysfunctional beliefs associated with that particular food. In contrast, blind weighing seemed to help break some of these links by providing limited or no weight information at all (*n* = 5).

#### Theme 3: treatment autonomy and team relationships

Within this theme, three subthemes were identified.

##### Treatment autonomy

Treatment autonomy was highly valued by participants across groups (*n* = 10). Participants expressed the belief that open weighing satisfies autonomy because it provides them with regular progress feedback (e.g., whether they are reaching their weight goals), evokes a sense of responsibility and ownership over their treatment, includes them in treatment conversations, and helps clarify changes to treatment (e.g., meal plan modifications). Open weighing also made participants feel as though they were “treated like a ‘normal person’ who could be trusted and could take responsibility” of their own treatment (R304). In comparison, blind weighing was associated with feeling “powerless” (BW107), “like a child” (R316) who could not be given responsibility, and excluded from treatment decisions.

##### Trust in treatment team

Trust in the treatment team was considered key for successful blind weighing (*n* = 14). Participants explained that, after establishing trust in their treating professionals, they were able to relinquish control over their weight. In contrast, when trust was compromised, participants reported continued self-weighing outside of the treatment program and increased treatment resistance.

##### Strategies for enhancing autonomy and trust

Participants highlighted that autonomy could be enhanced by providing a sense of choice over weighing practices. Participants strongly valued participation in treatment decision-making, including consultation about their views and preferences. Further, psychoeducation about the rationale for different treatment strategies (including weighing) and treatment decision-making transparency were viewed as important in facilitating patient cooperation and reducing anxiety. In this context, participants commented that alterations to blind weighing that included some weight feedback without disclosing actual numbers (e.g., whether or not the person had made a significant weight gain or loss of more than 1.5 kg) could help increase trust in the treatment team and explain (otherwise unexpected) changes to treatment (e.g., meal plan increases).

#### Theme 4: life outside of treatment

Within this theme, two subthemes were identified.

##### Weighing, weight exposure, and discharge support

Participants feared that exposure to their weight post-discharge would be confronting following blind weighing. Some participants deemed weighing to be “inevitable” (*n* = 4; e.g., in the context of a doctor’s appointment) and participants were concerned about their ability to cope and about possible relapse following weight confrontation on or post-discharge when they no longer had therapist support (*n* = 15). Continued blind weighing by the outpatient team on discharge from the inpatient program or gradual weight exposure throughout treatment were identified by participants as strategies to manage weight exposure post-discharge.

##### Weight change awareness

Participants reported that the number on the scale was not the only indicator of weight change (*n* = 7). Blind weighing participants expressed that, although they did not see the number when weighed, they were aware of shifts in their body weight based on fit of clothes and appearance in mirrors. Others found nonspecific feedback about their weight during blind weighing to be helpful to gain a sense of their progress (see Theme 5). Participants further elaborated that *nonspecific* weight information helped prevent unnecessary fixation on a *specific* number while providing enough information regarding weight change to prevent overt shock if they were incidentally weighed post-discharge.

#### Theme 5: weighing practice preferences and rationale

Within this theme, four subthemes were identified.

##### Preference

Weighing practice preferences differed with current treatment condition, although across all samples blind weighing was preferred by 58.5% (*n* = 24) of participants and open weighing by 36.6% (*n* = 15). Among recovered participants, 73.3% (*n* = 11) preferred blind weighing.

##### Open weighing

Participants who preferred open weighing (*n* = 15) expressed a strong “need” to know their weight, mainly to feel in control. Participants imagined or had previously experienced distress regarding “not knowing” their exact weight during blind weighing. Open weighing was reasoned to provide progress feedback, facilitate weight habituation, and help challenge exaggerated weight gain beliefs.

##### Blind weighing

Participants who preferred blind weighing (*n* = 23) thought that it relieves anxiety associated with weight change, shifts therapy from being predominantly weight-centred to focus on other aspects of the disorder and recovery, and facilitates body trust. Participants reported reduced engagement in eating disorder symptoms, less preoccupation with weight, and increased capability to engage in recovery. Further, some participants reported a preference for modified blind weighing (*n* = 6), that is the indication of weight changes without disclosing exact weight. Examples included the use of a numberless *weight graph* (graph depicts the weight trend but no specific numbers), *BMI banding* (patients are told when they move up or down a BMI band when their weight is in the “new band” for three consecutive readings), *significant weight gain or loss* (what is considered “significant” depends on the agreement between therapist and client), *meeting guideline* (confirmation whether a patient has met treatment guidelines regarding weekly weight gain), or *no gram information* (patient sees the kilograms on the scale but not the grams). Having an estimate of one’s weight was reported as helpful to challenging distorted cognitions about weight gain and providing a sense of security and control which facilitated trust in the treatment team and tolerance of weight uncertainty.

##### Stage of treatment

Participants’ weighing practice preference was related to stage of treatment. Blind weighing was rationalised to be most appropriate in the early, acute stages when the ED-Self is likely to be stronger than the Healthy-Self. Participants argued that blind weighing can shield patients from distress associated with weight change and thus improve therapy engagement and progress. At the same time, participants reflected on the importance of patients understanding the rationale for blind weighing, “being ready,” and trusting the treatment team for blind weighing to be effective. Conversely, open weighing was viewed as more suitable at later stages of recovery when patients are “in a better head space” and cognitive challenging has more potential to be effective.

### Quantitative findings

#### Weighing anxiety and weight concerns

Two separate Analyses of Variance (ANOVAs) were conducted to test for differences among the three samples (i.e., open weighing, blind weighing, recovered) in their reaction to being weighed (i.e., weight-related anxiety) and/or overall concern with weight. Group mean ratings of each variable are presented in Table 7. Results revealed a significant effect of sample on both weighing anxiety, *F* (2, 38) = 9.02, *p* = .001, *η*_*p*_^2^ = .32, and overall weight concerns, *F* (2, 38) = 47.77, *p* < .001, *η*_*p*_^2^ = .72. Post hoc comparisons using the Tukey HSD test indicated that mean weight-related anxiety for the open weighing group was significantly higher than it was for the blind weighing group (*p* = .004) and the recovered group (*p* < .001). The blind weighing group and the recovered group were not significantly different in weight-related anxiety (*p* = .339). Furthermore, recovered participants scored significantly lower on the weight concern scale than did both the blind weighing group (*p* < .001) and open weighing group (*p* < .001). There was no statistically significant difference on weight concerns between the two current patient groups (*p* = .275).

**Table 7:** Group Mean (SD) Ratings of Quantitative Variables

	OW *M* (*SD*)	BW *M* (*SD*)	Recovered *M* (*SD*)
Weight-Related Anxiety	86.48 (11.67)^a^	57.80 (25.87)^b^	48.97 (29.72)^b^
Weight Concerns	5.25 (0.75)^a^	4.86 (1.04)^a^	2.27 (0.84)^b^
• Intolerance of Weight Uncertainty	5.62 (0.65)^a^	4.31 (1.55)^b^	
• Weight Preoccupation	5.23 (1.09)	5.23 (0.93)	
• Weight Importance	5.13 (0.90)	4.92 (1.06)	

*Note.* OW: Open Weighing; BW: Blind Weighing. Means within a row with a different superscript are significantly different at *p* < .05. Subscale analyses for differences between current and recovered patients were not conducted

A follow-up two-tailed independent samples *t*-test was conducted to examine whether the two current patient samples (open weighing and blind weighing) differed in their scores on any of the three subscales comprising the weight concern scale.^2^ The results showed that open weighed patients reported significantly greater intolerance of weight uncertainty compared to blind weighed patients, *t*(24) = 2.81, *p* = .002, *d* = 1.10. Mean scores on the subscales, weight preoccupation and weight importance, were not significantly different.

## Discussion

Weighing is an established component of the treatment of eating disorders but there is little available data to indicate whether open or blind weighing is ideal. The current study explored how current and recovered eating disorder patients view and experience blind and open weighing practices. The findings provide insights into the acceptability of weighing practices and their perceived impact on therapy engagement and treatment outcomes.

In this study, most participants preferred blind weighing over open weighing. Consistent with eating disorder clinicians’ views [3], participants deemed blind weighing particularly beneficial in the acute stages of treatment when change ambivalence is most prominent. Participants thought that blind weighing decreased anxiety and eating disorder psychopathology (e.g., weight preoccupation, urges to engage in compensatory behaviours) and that blind weighing therefore increased their ability to engage and progress in treatment. In comparison, the majority of participants identified open weighing as a barrier to recovery, particularly throughout weight restoration, when seeing the number on the scale was thought to have negative effects on motivation, anxiety levels, urges to engage in compensatory behaviours, and treatment responsivity. Instead, participants saw merit in exposure to the number on the scale (i.e., open weighing) when in a better headspace during the later stages of recovery because it allowed them to challenge distorted cognitions regarding their weight. Patients’ accounts of increased distress when exposed to their weight were supported by quantitative findings in this study in which open weighing was associated with significantly greater weight-related anxiety than blind weighing. Based on the current findings, withholding weight information early in treatment may be beneficial as a means of reducing weight-related anxiety and maximising treatment engagement. Early response to treatment can help prevent premature treatment dropout [17] and is predictive of better outcomes [18]. Thus, at least in the early stages of treatment, it makes conceptual sense to use a weighing practice that mitigates patients’ distress and facilitates treatment responsivity. However, it is conceivable that, at least for some patients, blind weighing represents a safety behaviour which allows patients to avoid some of their fears (e.g., knowing their weight) and interfere with long-term treatment outcomes. Thus, it is important to consider that different approaches to weighing might be suited at different stages of treatment. For example, blind weighing early in treatment might help patients engage more thoroughly in other aspects of the treatment (e.g., adhering to meal plan), but transitioning to open weighing when patients are less acute (e.g., in an outpatient setting) may be preferable in order to challenge the “broken cognition”, extinguish the patient’s fears of being weighed and help patients cope with being a certain weight.

In reference to weight-related anxiety, the findings of the present study stand in contrast to the habituation effect postulated for open weighing which assumes that patients experience a reduction in weight-related anxiety following repeated exposure to their weight [1]. There may be several possible explanations for the current findings. For example, as alluded to by Forbush and colleagues [3], open weighing is only effective for decreasing weight-related anxiety when all maladaptive coping strategies and responses (e.g., increased eating disorder behaviours following weighing) are blocked. Given that the patient sample in the current study consisted of women who were very acute and therefore required inpatient care, they may not have been able to resist engaging in some eating disorder behaviours during their admission, thereby rendering weight exposure ineffective and potentially explaining the high level of anxiety symptoms in the open weighing group. An alternative explanation for the current findings is that the inclusion criteria used for the patient sample in the current study (i.e., completion of two or more weeks of inpatient treatment) possibly meant that some patients had not been in inpatient treatment for long enough to experience the habituation effect. However, if weight-related anxiety negatively affects treatment retention as suggested by participants in this study, it may be that patients drop out before habituation can occur. Future research is needed to address these issues.

The theme of control and intolerance of uncertainty emerged in most interviews. Participants who had experienced blind weighing described initially having significant difficulties adjusting to weight ambiguity, noting that not knowing their weight was a threat to their sense of control. However, participants viewed this need for control to be pathological (i.e., ED-Self driven) and consequently reasoned that learning to tolerate uncertainty around one’s weight is a necessary step towards recovery from an eating disorder and living a normal, healthy life. This is consistent with existing literature recognising that patients’ readiness to let go of pathological control is crucial to treatment success [19–21]. Blind weighing was perceived to provide an opportunity to modify the distorted beliefs regarding the need to know and control one’s weight. Supporting participants’ accounts, quantitative analyses found that blind weighed patients had greater tolerance of weight uncertainty than did open weighed patients. These results suggest that blind weighing could have clinical utility in that it (1) reduces weight-related anxiety and maximises engagement, and (2) successfully exposes patients to uncertainty and lack of control over their weight, facilitating a shift in maladaptive beliefs regarding the dangers of uncertainty or lack of control (e.g., “I must know my exact weight at all times”). An interesting finding was the suggestion of using blind weighing adaptations that provide some level of weight feedback without disclosing “exact” weight information. Participants perceived these alternative approaches to satisfy their need for certainty and control without reinforcing the obsession over the numeric value. These findings suggest that weighing adaptations may be promising in lowering the perceived barrier to blind weighing (i.e., perceived lack of control over weight). Empirical research is required to determine the precise benefits of these adaptations and explore the impact they have on treatment outcomes.

Almost uniformly, participants valued autonomy and involvement in treatment decision-making. Autonomy could be experienced two ways: by retaining control of one’s weight or by choosing to let go of that control, as long as the decision was made autonomously by the participant. A trusting and collaborative therapeutic relationship appeared to enable participants to relinquish control over their weight and adhere to the weighing practice guidelines (i.e., not to self-weigh outside of treatment). In contrast, when the therapeutic relationship was precarious, participants reported poorer adherence to blind weighing guidelines (i.e., not weighing themselves outside of treatment), which they thought negatively affected their recovery progress. These findings are consistent with research showing that patients who comprehend the treatment process and feel that their need for autonomy is met by being involved in important treatment choices are less likely to drop out of treatment [22] and also achieve improved treatment motivation, engagement, and outcomes [23, 24] than do patients whose autonomy needs are not supported as strongly. Thus, whatever weighing practice is used, clinicians should ensure that patients are consulted and involved as much as possible.

Finally, participants expressed concerns regarding incidental weight exposure post-discharge after not having seen their weight during their admission. As it stands, research acknowledges that, in eating disorders, the transition from inpatient treatment to home is often problematic and associated with a high risk of relapse [25–27]. To minimise risk of readmission, it is recommended that a continuous stepped care approach, including a post-discharge support system, be provided [26, 28]. This may be particularly important for blind weighed patients who have not been aware of their weight throughout the inpatient admission and thus are at risk of relapsing if exposed to their weight prematurely. To explore the possible link between blind weighing and relapse, longitudinal research comparing patient trajectories after blind versus open weighing is needed. Furthermore, future research may benefit from investigating how patients’ transition between different levels of care (e.g., from inpatient to outpatient services) can be improved to be as seamless and supportive as possible.

Limitations of this study include the “opt-in” nature of recruitment and potential for self-selection bias. Thus, findings may reflect the views of people with eating disorders who are more interested in the debate about weighing practices. Second, the current patient group consisted exclusively of female adults with severe eating disorders (i.e., requiring inpatient care), the majority of whom met criteria for AN. Therefore, this study might not be representative of the wider spectrum of eating disorders or those individuals who receive outpatient treatment. The recovered participants, however, included women who had experienced both inpatient and outpatient care thus speaking to the generalisability of the findings. Third, current blind weighed patients and recovered participants differed significantly in age and BMI from current open weighed patients. Furthermore, information regarding duration of illness and time in treatment were not collected. It is conceivable that the groups’ experiences and opinions would vary with these factors. Finally, the current study focused on patient perspectives rather than on treatment outcomes. Patient perspectives can help maximise therapy engagement and, consequently, retention (both of which are particularly difficult when treating eating disorder patients), however future randomised controlled trials comparing the two weighing practices are necessary to determine how open and blind weighing influence treatment effectiveness. Ideally these trials would include qualitative assessments of patient’s experience so that the direct link between treatment effectiveness and experience can be mapped out.

## Conclusions

Qualitative research allows exploration and understanding of lived experience and is ideally suited to expanding the research base on weighing practices in eating disorders. Overall the findings of the current study suggest that both open weighing and blind weighing have clinical utility. Blind weighing appears to be preferred by patients, especially in the acute stages of treatment, because it reduces weight-related anxiety and increases patients’ ability to engage in recovery. In addition, blind weighing is viewed to promote tolerance of weight uncertainty. In comparison, open weighing is perceived to be more beneficial in later stages of treatment. Although informative, this was a qualitative study, and clinical practice should not be guided by patients’ perspectives alone. Thus, experimental designs comparing the two weighing practices are needed before definitive conclusions can be drawn. The next step for future research will be to connect the patient insights gained from the current study with treatment outcomes gained from randomised controlled trials in order to develop treatment protocols that maximise effectiveness, as well as patient engagement and retention.

## Supplementary information

**Additional file 1.** Interview Guide for open and blind weighed participants.

**Additional file 2.** Interview Guide for recovered participants.

## Data Availability

The datasets used and/or analysed during the current study are available from the corresponding author on reasonable request.

## Abbreviations

AN: Anorexia nervosa
ANOVA: Analysis of Variance
BMI: Body mass index
BN: Bulimia nervosa
BW: Blind weighing
CBT: Cognitive-behaviour therapy
DSM-5: Diagnostic and Statistical Manual of Mental Disorder, Fifth Edition
EDDS: Eating Disorder Diagnostic Scale – DSM-5 version
ED-Self: Eating disorder-self
FBT: Family-based treatment
OSFED: Other specified feeding or eating disorders
OW: Open weighing

## Footnotes

1 Codes associated with particular responses reflect the following: BW, blind weighed inpatient; OW, open weighed inpatient; R, recovered participant; #, participant number.

2 Note that analyses of differences on weight concern subscales were only conducted for current patient groups to determine whether the two weighing practices had a differential effect on these variables. The majority of recovered participants (53.3%) had been both open and blind weighed throughout their treatment and so changes on these variables in the recovered group could not be attributed to a specific weighing practice.
