# Supplementary material for: Blind versus open weighing from an eating disorder patient perspective
Source: J Eat Disord. 2020 Aug 17;8:39. doi: 10.1186/s40337-020-00316-1 (PMC7429892; doi:10.1186/s40337-020-00316-1)
Supplement: Supplementary file 1 — Additional file 1. Interview Guide for open and blind weighed participants. [file 40337_2020_316_MOESM1_ESM.docx]

**Additional File 1: Clinical Interview Items, Current Patients^[[1]](#footnote-1)^**

1. **General weighing behaviour**
2. Before you were admitted to the clinic, how often did you use to weigh yourself?
3. Has it always been like that or have you ever weighed yourself more or less frequently? Can you recall why that might have been?
4. **Patient preferences**
5. At this clinic, staff don’t share your weight information with you. How are you finding it?
6. Would you prefer that staff told you your weight or would you rather not know? Why?
7. What type of weighing do you think would be better for your treatment and why?
8. Have you ever been in a facility where they do open weighing? How did you find that?
9. Do you think that being blind weighed influences how motivated you are with treatment? If so, how?
10. Do you think that being blind weighed affects how well you can stick to your meal plan? How so?
11. Does not knowing your daily weight affect your preoccupation with your weight? If so, how?
12. How do you feel the day before being weighed? What about on weighing days? How do you feel after having been weighed?
13. Does not seeing you weight have an impact on your eating disorder symptoms? If so, how?
14. Does not knowing your weight in any way affect your engagement in compensatory behaviours (e.g., excessive exercise, refused meal plan, self-induced vomiting)? Would it be different if you were told your weigh on a regular basis?
15. **General opinion**
16. Let’s talk about it more generally.
17. In your opinion, are there any benefits to blind weighing? What are some drawbacks? What about open weighing?
18. At what stage of treatment, if ever, do you think that patients should know their weight?

1. The questions below are framed for a clinic that uses blind weighing. Parallel questions were asked for a clinic that uses open weighing. [↑](#footnote-ref-1)
