# Supplementary material for: Blind versus open weighing from an eating disorder patient perspective
Source: J Eat Disord. 2020 Aug 17;8:39. doi: 10.1186/s40337-020-00316-1 (PMC7429892; doi:10.1186/s40337-020-00316-1)
Supplement: Supplementary file 2 — Additional file 2. Interview Guide for recovered participants. [file 40337_2020_316_MOESM2_ESM.docx]

**Additional File 2: Clinical Interview Items, Recovered Patients**

1. **General questions about treatment**
2. When were you in treatment for your eating disorder?
3. What type of treatment was it (inpatient, outpatient, individual therapy)? Roughly, how long for?
4. Do you know what type of eating disorder you were diagnosed with?
5. **General weighing behaviour**
6. Do you remember how often you used to weigh yourself before beginning treatment?
7. **Patient preferences**
8. Did you know your weight during therapy or not? [If no] When did you find out (if ever)? How did you feel finding out?
9. How did you find it not knowing your weight during therapy?
10. Would you have preferred that staff told you your weight or did you rather not know? Why?
11. What type of weighing do you think would have been for your treatment and why?
12. Have you ever been in a facility where they do open weighing? How did you find that?
13. Do you think that being blind weighed influenced how motivated you were with treatment? If so, how?
14. Do you think that being blind weighed affected how well you could stick to your meal plan? How so?
15. Did not knowing your daily weight affect your preoccupation with your weight? If so, how?
16. Can you recall how you felt on weighing days? What about the days leading up to it/afterwards?
17. Did not seeing you weight have an impact on your eating disorder symptoms? If so, how?
18. Did not knowing your weight affect your engagement in compensatory behaviours (e.g., excessive exercise, refused meal plan, self-induced vomiting)? Would it be different if you were told your weigh on a regular basis?
19. How has it been since? How often do you weigh yourself now?
20. **General opinion**
21. Let’s talk about it more generally.
22. In your opinion, are there any benefits to blind weighing? What are some drawbacks? What about open weighing?
23. At what stage of treatment, if ever, do you think that patients should know their weight?
24. Is there anything else that you think researchers should know about this topic?
